# Multi-omics analyses unveil gut microbiota and metabolites signatures in deoxycholic acid-associated intestinal inflammation

**DOI:** 10.3389/fmicb.2026.1917982

**Published:** 2026-09-03

**Authors:** Gaojie Zhang, Ye Huang, Zizhen Gong, Congfeng Xu, Xianglin Ding, Wei Cai, Jin Wu

**Affiliations:** 1 Department of Pediatric Surgery, Xinhua Hospital, School of Medicine, Shanghai Jiaotong University, Shanghai, China; 2 Shanghai Institute for Pediatric Research, School of Medicine, Shanghai Jiaotong University, Shanghai, China; 3 Shanghai Key Laboratory of Pediatric Gastroenterology and Nutrition, Shanghai, China; 4 Department of Cardiology, Shanghai Jiao Tong University Affiliated Sixth People's Hospital, Shanghai, China; 5 Department of Gastroenterology, Suzhou Yongding Hospital, Suzhou, Jiangsu, China

**Keywords:** deoxycholic acid, gut microbiota, high-fat diet, intestinal inflammation, metabolomics

## Abstract

**Objective:**

High-fat diet (HFD) is closely related to the increased incidence of inflammatory bowel disease (IBD), and excessive fecal deoxycholic acid (DCA) induced by HFD makes significant contribution to the colonic inflammation. However, the precise mechanisms remain unclear. This study aims to explore the association between DCA-induced alteration of gut microbiota as well as related metabolites and intestinal inflammation.

**Methods:**

Wild-type C57BL/6 J mice were orally administrated with or without 0.2% DCA for 12 weeks, then the alteration of gut microbiota signature and fecal metabolites were analyzed by metagenomic sequencing and widely-targeted metabolomics, respectively. The colonic tissue injury was confirmed by histopathological analysis and pro-inflammatory cytokines production was determined by qPCR and ELISA.

**Results:**

DCA administration induced gut microbiota dysbiosis and fecal metabolomic profile disturbance, accompanied with significantly increased expression of pro-inflammatory cytokines in intestine, including TNF-*α*, IL-6 and IL-1β, and obvious tissue damage. Specifically, α-diversity of gut microbiota was greatly reduced by excessive DCA, and abundance analysis together with linear discriminant analysis of effect size (LEfSe) identified *Bacteroides* and *Desulfovibrio* as potential biomarkers of DCA exposure. Excessive DCA significantly decreased the abundance of *Eubacterium plexicaudatum*, *bacterium 1xD42-87* and *Ruminococcus flavefaciens*, which were positively correlated with the downregulation of multiple metabolites reported to possess anti-inflammatory activities, especially indoles, vitamin D3 and alpha-CEHC. Meanwhile, DCA administration dramatically increased the abundance of *Parabacteroides distasonis* and *Bacteroide acidifacien*, which were positively correlated with the upregulation of metabolites reported to have pro-inflammatory properties, including multiple bile acid metabolites such as chenodeoxycholic acid, glycochenodeoxycholic acid and lithocholic acid. Spearman correlation analysis emphasized the important effects of aforementioned microbiota and metabolites in the association between DCA and intestinal inflammation.

**Conclusion:**

Our study revealed that excessive DCA led to concurrent alterations of gut microbiota and metabolites, which exhibited significant correlations with intestinal inflammation, suggesting a potential indirect regulatory pathway that may involve gut microbiota. Targeting DCA-related gut microbiota or metabolites might represent a promising intervention for HFD-associated colonic inflammation.

## 1 Introduction

Consumption of westernized high-fat diet (HFD) is closely associated with diverse inflammatory disorders, involving type 2 diabetes, cardiovascular disease and IBD, which commonly attributes to the induction of colonic and subsequent systemic low-grade inflammation (Duan et al., 2018; Li et al., 2020; Nabhani et al., 2019; Dang et al., 2023). Accumulating evidence discloses that long-term HFD intake positively correlates with the increased risk of both ulcerative colitis and Crohn’s disease, especially highlighting the critical role of HFD-induced dysregulation of intestinal bile acid metabolism (Stenman et al., 2013a; Stenman et al., 2013b; Devkota and Chang, 2015; Liu et al., 2023).

Deoxycholic acid (DCA), a major colonic secondary bile acid, has attracted increasing attention on the occurrence of IBD under HFD settings (Xu et al., 2021; Ju et al., 2023; Liu et al., 2021). A high-fat diet could stimulate the synthesis of primary bile acids (PBAs) and upregulate the abundance of key gut microbiota involved in the transformation of PBAs into secondary bile acids (SBAs) in the colon, thus leading to the increased production of SBAs, especially DCA. Prolonged HFD exposure results in an approximate ten-fold increase in colonic DCA levels (Stenman et al., 2012), and mice fed with DCA-supplemented diet could develop obvious colonic inflammation similar to human IBD. Furthermore, DCA also markedly exacerbates experimental colitis induced by DSS or TNBS (Zhao et al., 2016; Li et al., 2023). However, the mechanisms underlining the association of gut inflammation with DCA still remain elusive.

Intestinal tract residents trillions of microbes, and microbiota together with their metabolites have long been recognized as integral components in orchestrating intestinal homeostasis (Li T. et al., 2024; Wu et al., 2024; Wang J. et al., 2024; Lin and Zhang, 2017). Gut microbiota regulates bile acid (BA) metabolism, and BAs can reshape the microbial community conversely. HFD-induced excessive DCA in intestine may affect the composition, diversity as well as metabolic activity of microbiota, which can further lead to the alteration of multiple microbial metabolites, such as short chain fatty acid and indole derivatives, eventually contributing to the initiation of gut inflammation. A comprehensive exploration is necessary to understand how DCA creates an intestinal pro-inflammatory environment by manipulating gut microbiota and related metabolites.

In the present study, extensive analysis combining microbial metagenomic sequencing and widely-targeted metabolomics was conducted to evaluate changes in the gut microbiota and metabolites of DCA-administrated mice compared to the controls. Our study suggests that excessive DCA led to the significant increase in the abundance of *P. distasonis* and *B. acidifaciens* species and dramatic decrease in the abundance of *E. plexicaudatum*, *bacterium 1xD42-87* and *R. flavefaciens* species, which are closely associated with the up-regulation of bile acid metabolites reported to possess pro-inflammatory activities along with the down-regulation of metabolites reported to hold anti-inflammatory activities, involving indoles, vitamin and hormone-like metabolites in intestine, therefore indicating an potential correlation with the occurrence of gut inflammation. These findings could be beneficial to the comprehensive understanding of the mechanisms concerning HFD-induced gut inflammation and identify new therapeutic targets.

## 2 Materials and methods

### 2.1 Animal experiments

Male C57BL/6 J mice (8-week-old) were purchased from Shanghai Jihui Laboratory Animal Care Co., Ltd. (Shanghai, China) and housed in the SPF animal facility under standard temperature/humidity conditions and a 12-h light/12-h dark cycle. All the experiment procedures were performed in accordance with the Guide for the Care and Use of Medical Laboratory Animals issued by the Ministry of Health of China and approved by the Animal Care and Use Committee of Xinhua Hospital Affiliated to Shanghai Jiao Tong University. Mice were randomly assigned into control (CON) and DCA-administrated (DCA) groups (*n* = 5 per group). After one week of acclimation (defined as week 0), mice in the DCA group received 0.2% DCA (Sigma, D6750) in the drinking water ad libitum for 12 weeks. Body weight was recorded weekly and mice were euthanized at 13th week for sample collection. For euthanasia, mice were anesthetized by intraperitoneal injection of 1% sodium pentobarbital at 50 mg/kg. After adequate anesthesia was confirmed, the mice were then euthanized by cervical dislocation. The paraffin sections of colon tissues were used for hematoxylin and eosin (H&E) staining and colon homogenates were prepared for inflammatory cytokines detection.

The DCA concentration (0.2%) used in our *in vivo* study was determined based on the previous research. Bernstein et al., fed the mice a diet supplemented with 0.2% DCA to establish a colitis mouse model simulating HFD (Bernstein et al., 2006). Thereafter, 0.2% DCA regimen in drinking water or diet has been widely adopted for inducing sustained luminal DCA exposure in numerous studies related to gut inflammation and gastrointestinal cancer (Wang et al., 2020; Huang et al., 2023; Liu et al., 2025). The 12-week exposure duration was also selected based on the published studies. 12-week regimen at the same drinking-water concentration of DCA (0.2%) has been proved to increase intestinal permeability, alter tight-junction protein expression and raise colonic inflammation (Pan et al., 2024). Liu et al. also reported a 12-week drinking-water study (0.2% DCA) in Apc^Min/+^ mice that induced barrier impairment and low-grade inflammation (Liu et al., 2018).

Fecal samples were obtained freshly for microbial metagenomic sequencing and widely-targeted metabolomics. Briefly, after a 12-week DCA intervention, each mouse was individually placed in a clean, sterilized collection container. Several freshly voided fecal pellets were collected promptly after defecation using sterile forceps and transferred into a sterile microcentrifuge tube as a single fecal sample. Each sample was divided into two portions, one for widely-targeted metabolomic profiling and the other for shotgun metagenomic sequencing. Both portions were immediately placed on dry ice and subsequently stored at −80 °C until analysis.

### 2.2 Histopathological analysis

Colonic tissue histopathological scoring was performed as described previously in a blinded manner (Heazlewood et al., 2008), including crypt architecture, crypt length, crypt abscesses, tissue damage, goblet cell loss, inflammatory cell infiltration and lamina propria neutrophils. The combined histopathological score ranged from 0 to 24.

### 2.3 Quantitative real-time PCR

To examine gene expression of pro-inflammatory cytokines in murine colon, total RNA was isolated from colon tissues using an EZB RNA extraction kit (EZB) according to the manufacturer’s instructions. RNA was then reverse-transcribed into cDNA by a Vazyme reverse transcription kit (Vazyme). Quantitative PCR was performed using a SYBR Green-based qPCR master mix (Vazyme) on the 7,300 Real-Time PCR System (Applied Biosystems). Primer sequences were as follows: TNF-*α*, forward 5′- GGT GCC TAT GTC TCA GCC TCT T − 3′ and reverse 5′- GCC ATA GAA CTG ATG AGA GGG AG -3′; IL-6, forward, 5′-TAG TCC TTC CTA CCC CAA TTT CC-3′ and reverse, 5′-TTG GTC CTT AGC CAC TCC TTC-3′; IL-1*β*, forward, 5′- TGG ACC TTC CAG GAT GAG GAC A-3′ and reverse, 5′- GTT CAT CTC GGA GCC TGT AGT G-3′; β-actin, forward, 5′- GGC TGT ATT CCC CTC CAT CG − 3′ and reverse, 5′- CCA GTT GGT AAC AAT GCC ATG T − 3′.

### 2.4 Enzyme-linked immunosorbent assay (ELISA)

To confirm changes in protein levels of pro-inflammatory cytokines in murine colon, colonic tissues were harvested and briefly rinsed. Colon homogenates were prepared in 200 μL ice-cold PBS supplemented with protease inhibitor and phosphatase inhibitor. Homogenates were then sonicated on ice followed by centrifugation at 12,000 rpm for 10 min at 4 °C. Supernatants were collected for detection of IL-1β, TNF-*α* and IL-6 levels by ELISA kits (Lianke, China) according to the manufacturer’s instructions. Cytokine concentrations were calculated based on standard curves and normalized to corresponding total protein concentrations determined by a BCA assay.

### 2.5 Widely-targeted metabolomics profiling

20 mg of feces per sample were extracted with 400 μL of 70% methanol in water containing internal standards, sonicated for 10 min, incubated and then centrifuged at 12,000 rpm. After repeated transfer and centrifugation, 200 μL supernatant was placed into an auto-sampler vial insert for instrumental analysis. Chromatographic separation was performed on a Waters ACQUITY UPLC HSS T3 C18 column (1.8 μm, 2.1 mm × 100 mm), and hydrophilic interaction liquid chromatography was conducted with a Waters ACQUITY UPLC BEH hydrophilic interaction liquid chromatography column (1.7 μm, 1 mm × 100 mm). Using multiple reaction monitoring, metabolites were quantified across all samples on an ExionLC AD system coupled to an AB SCIEX QTRAP 6500 tandem mass spectrometry platform. Peak integration and correction were performed using MultiQuant software, and peak areas were used as relative metabolite abundances. Differently expressed metabolites were determined according to the criteria of *p* < 0.05, and fold change > 2 or < 0.5 between the DCA and control groups. Metabolite identities were then cross-validated using HMDB, ChEBI, PubChem, and METLIN databases. Since the present study aims to identify metabolites that may be functionally relevant to DCA exposure-triggered intestinal inflammation, we prioritized for downstream correlation analysis those differential metabolites that met the following criteria: previously documented involvement in inflammatory responses retrieved from PubMed/MEDLINE, and directional changes consistent with the pro-inflammatory phenotype of our DCA exposure model, i.e., elevated pro-inflammatory metabolites or decreased anti-inflammatory metabolites.

### 2.6 Shotgun metagenomic sequencing and analysis

Total DNA was extracted from fecal samples using the VAMNE Magnetic Stool/Soil DNA Extraction Kit (Vazyme Biotech Co., Ltd., Nanjing, China; Cat. No. DMA5103-01), which combines chemical and mechanical lysis with magnetic-bead-based purification. Mechanical disruption was performed in the kit’s zirconia bead tubes using a JX-48 tissue grinder (Shanghai Jingxin Industrial Development Co., Ltd., Shanghai, China), and DNA purification was performed using a VNP-32P automated nucleic acid extraction instrument (Vazyme Biotech Co., Ltd.) according to the manufacturer’s protocol. DNA concentration was measured with a Qubit 4.0 fluorometer (Thermo Fisher Scientific, Waltham, MA, USA) and integrity was assessed with a Qsep400 Bio-Fragment Analyzer (BiOptic Inc., New Taipei City, Taiwan).

Sequencing libraries were constructed from 200 ng DNA per sample using the VAHTS Universal Plus DNA Library Prep Kit for Illumina V2 (Vazyme Biotech Co., Ltd.; Cat. No. NDB627-01) according to the manufacturer’s instructions, comprising enzymatic fragmentation with integrated end-repair and dA-tailing, followed by adapter ligation and PCR amplification (5 cycles), according to the manufacturer’s instructions, with DNA fragmentation targeting approximately 300 bp. Libraries were quantified with the Qubit 4.0 and insert-size distributions were assessed with the Qsep400; libraries passing QC were pooled and sequenced as 150-bp paired-end reads on an Illumina NovaSeq X Plus System. Host-derived reads were removed by aligning raw paired-end reads to the *Mus musculus* GRCm39 primary assembly (Ensembl release 109) using Bowtie2 v2.3.4 with the parameters --end-to-end --sensitive -I 200 -X 400 and removing reads mapped to the host genome. The remaining reads were processed with fastp v0.23.4 for adapter trimming and quality filtering. Reads were discarded when >40% of bases had Phred quality scores < 15, when they contained >5 ambiguous bases (N), or when their length after trimming was < 15 bp. The retained high-quality, non-host reads were used for metagenomic assembly, gene prediction, taxonomic profiling, and functional annotation. Taxonomic profiling was performed by DIAMOND blastp alignment of unigene protein sequences against the NCBI nonredundant database (Version 2022.05), followed by lowest common ancestor assignment using MEGAN. Functional annotation was conducted against commonly used databases including Kyoto Encyclopedia of Genes and Genomes, eggNOG and Gene Ontology. Between-group differential analysis of taxonomic features was performed by Metastats with multiple testing correction using the Benjamini–Hochberg method, and linear discriminant analysis effect size (LEfSe) was applied using Kruskal–Wallis and Wilcoxon rank-sum tests. Differential gene abundance analysis was performed applying DESeq2 on raw read counts with Benjamini–Hochberg false discovery rate correction (absolute log2-fold change greater than 2 and false discovery rate below 0.05).

### 2.7 Statistical analysis

All data were expressed as mean ± standard error of the mean (s.e.m.). Data analysis was performed using Graph Pad 9.5 software. Statistical significance between two groups was assessed by unpaired 2-tailed *t*-test with Welch’s correction or Mann–Whitney *U* test for the nonparametric data. Differences were considered to be statistically significant for *p* < 0.05.

## 3 Results

### 3.1 DCA reduces gut microbiota diversity and alters microbial community composition

**Figure 1:**
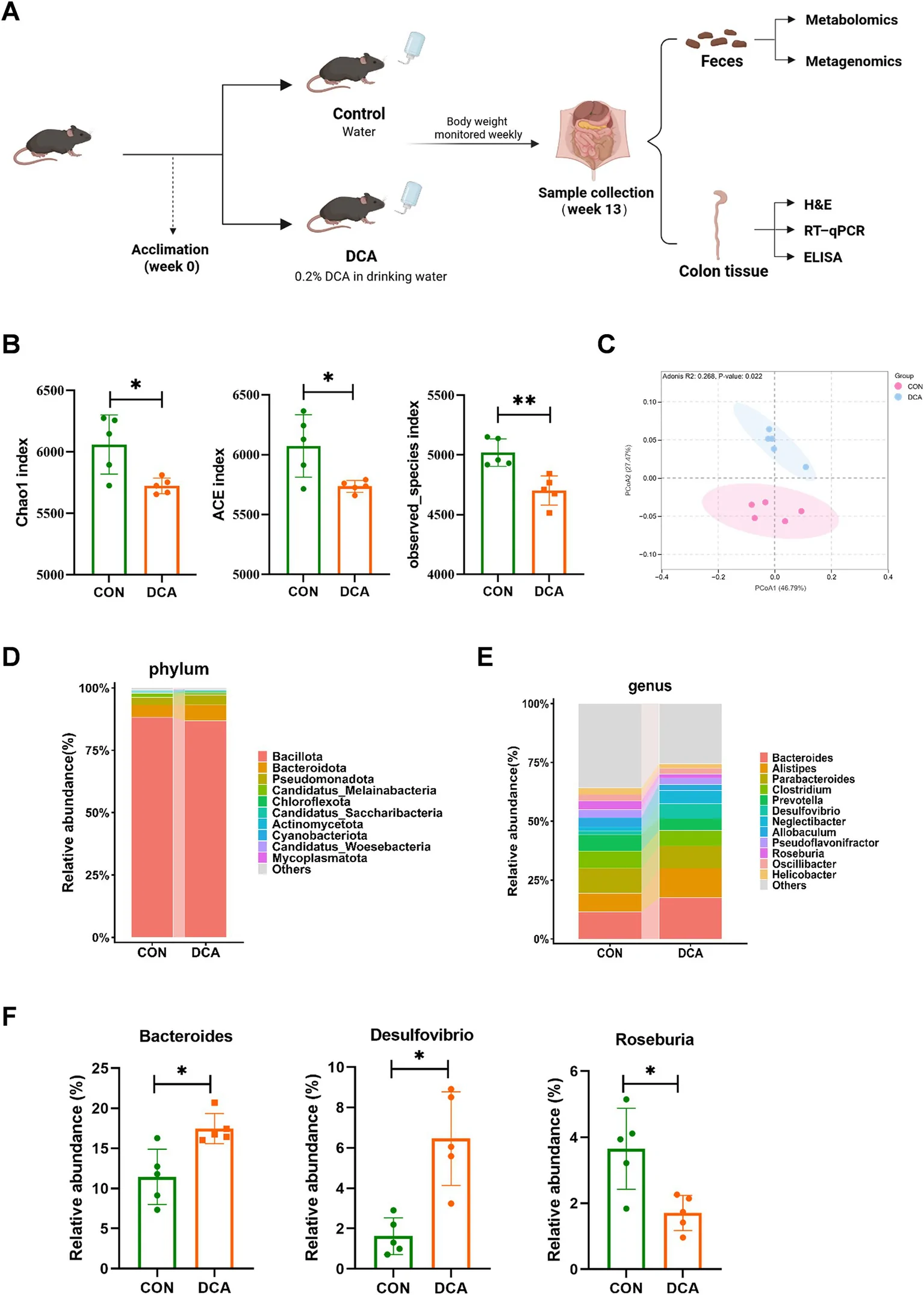
DCA reduces gut microbiota diversity and alters microbial community composition. **(A)** Animal experiment procedure (*n* = 5 per group). **(B)** Alpha diversity assessed using Chao1, ACE and observed_species index (*n* = 5). **(C)** Beta diversity presented by Principal coordinate analysis (PCoA). **(D)** Relative abundance of bacteria at the phylum level. **(E)** Relative abundance of bacteria at the genus level. **(F)** Histograms of gut microbiota with differential abundance at the genus level between two groups. *p < 0.05; **p < 0.01.

Considering the importance of intestinal microbiome in the gut homeostasis, wild-type C57BL/6 mice were orally administrated DCA for 12 weeks and the impact of DCA on the gut microbiota diversity and composition was explored through metagenomic sequencing in feces (Figure 1A). The *α*-diversity was significantly reduced in the DCA-treated group compared with the control, as evidenced by the Chao1, ACE and observed species index (Figure 1B). The PCoA plot exhibited distinct clustering patterns between the control and DCA groups (Figure 1C). Abundance analysis showed that, at the phylum level, the intestinal microbes of the two groups mainly consisted of *Bacillota* (formerly *Firmicutes*), *Bacteroidota* (formerly *Bacteroidetes*) and *Pseudomonadota* (formerly *Proteobacteria*) (Figure 1D). At the genus level, the relative abundances of *Bacteroides* and *Desulfovibrio* were significantly elevated in DCA-treated group compared with the control (*p* = 0.022 and *p* = 0.012 respectively), while the relative abundance of *Roseburia* was remarkably decreased in the DCA group (*p* = 0.037) (Figures 1E,F).

**Figure 2:**
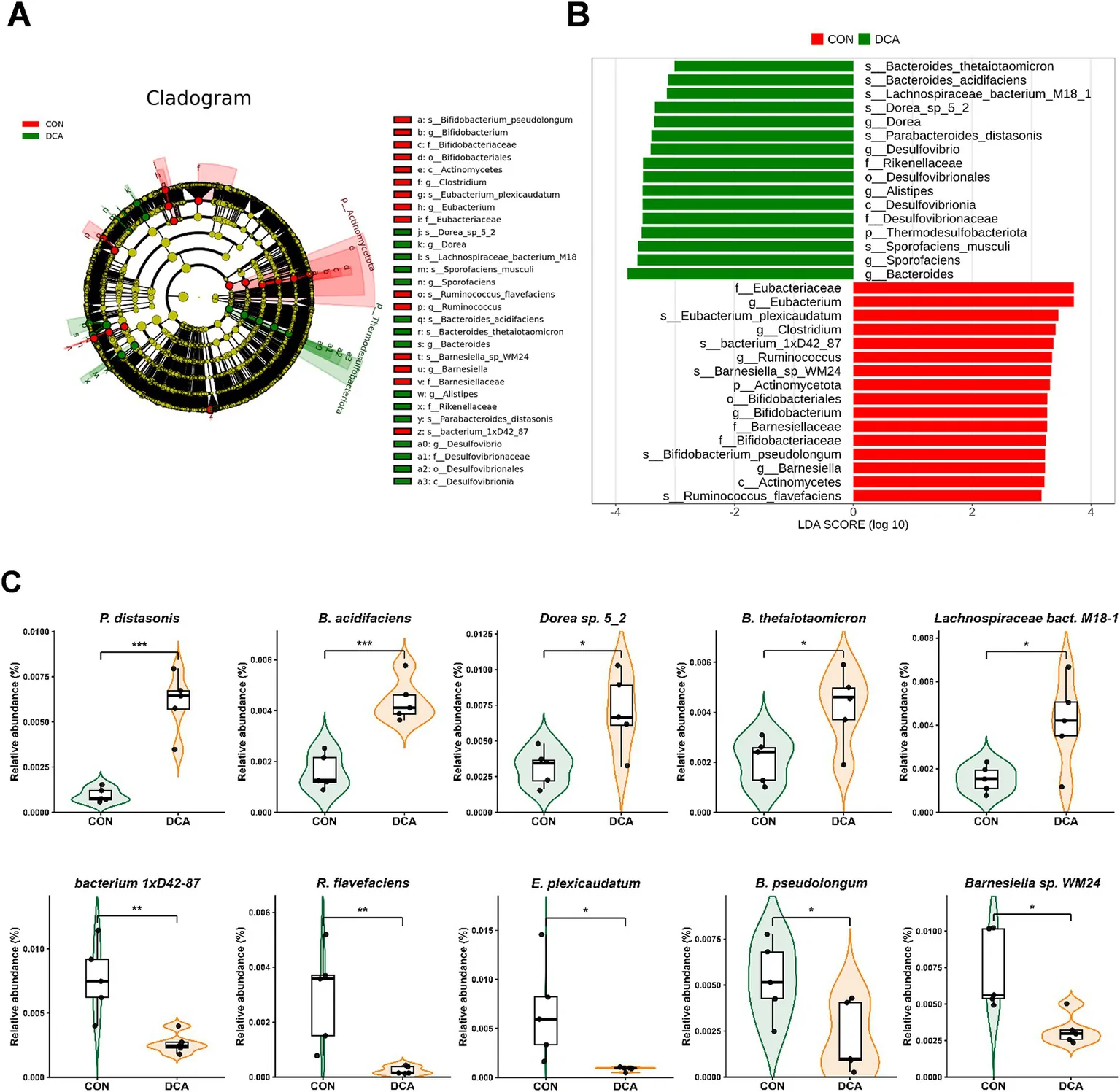
LEfse analysis of gut microbiota in control and DCA-treated mice. **(A)** Cladogram displaying the microbial community profiles in control (red) and DCA group (green). **(B)** LDA value distribution histogram (LDA > 3). **(C)** Histograms of 10 candidate microbial species with differential abundance in control and DCA groups. *p < 0.05; **p < 0.01; ***p < 0.001.

The microbial variance between the control (CON) and DCA groups was further analyzed by linear discriminant analysis (LDA) effect size (LEfSe) (LDA > 3, Figure 2A) to identify biomarkers in the DCA-treated group. Results exhibited that genera such as *Bacteroides*, *Desulfovibrio*, *Dorea*, *Sporofaciens* and *Alistipes* were enriched in the DCA group, while *Eubacterium*, *Ruminococcus*, *Barnesiella*, *clostridium* and *Bifidobacterium* were enriched in the CON group (Figure 2B). Abundance analysis together with LEfSe suggest that *Bacteroides* and *Desulfovibrio* may serve as potential biomarkers of DCA exposure, and genera enriched in the CON group are mainly associated with intestinal homeostasis maintaining. At the species level, a total of 11 differential abundant bacteria were annotated. Ten of these species with previously documented biological relevance to intestinal inflammation were retained for the subsequent correlation analysis. *Sporofaciens musculi* was not included, because published functional animal experiments indicated that *Sporofaciens musculi* showed no obvious effect on experimental colitis (Wang Y. et al., 2022). Among them, *P. distasonis*, *B. acidifaciens*, *Lachnospiraceae bacterium M18-1*, *Dorea* sp. *5_2* and *Bacteroides thetaiotaomicron* were enriched in the DCA group, whereas *Bifidobacterium pseudolongum, E. plexicaudatum*, *bacterium 1xD42-87*, *R. flavefaciens* and *Barnesiella sp_WM24* were enriched in the CON group with most of which possessing anti-inflammatory activity (Figure 2C). These results indicate that the excessive DCA contributes to gut microbiota dysbiosis.

### 3.2 DCA disturbs fecal metabolomic profile

**Figure 3:**
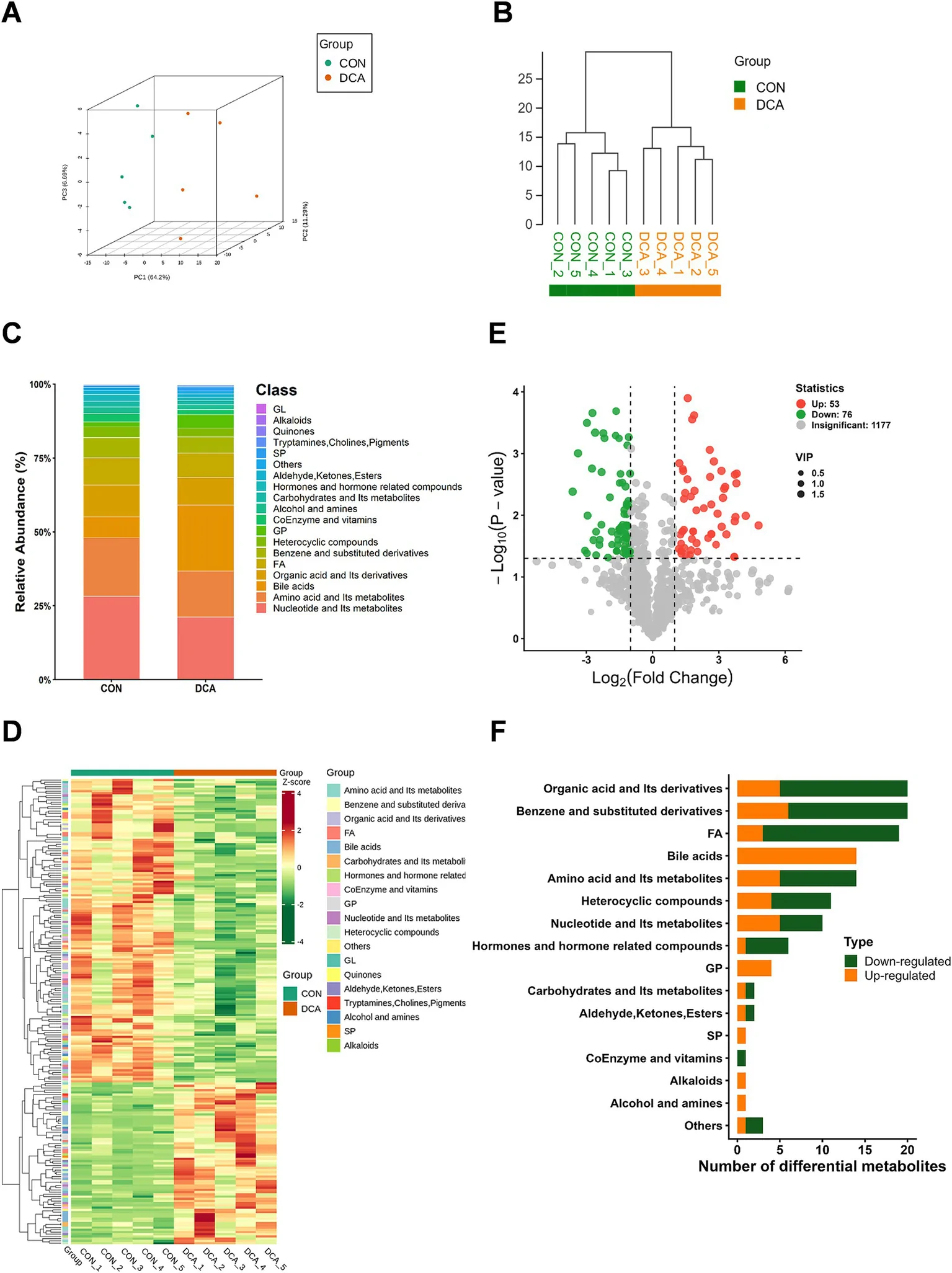
DCA disturbs fecal metabolomic profile **(A)** Unsupervised PCA showing significant differences between control and DCA groups (*n* = 5). **(B)** Hierarchical clustering tree of samples. **(C)** Relative abundance of the metabolites between control and DCA group. **(D)** Cluster heatmap showing the different metabolites in two groups. **(E)** Volcano plot of the differentially expressed metabolites in control and DCA groups. Red dots represent significantly upregulated metabolites, and green dots indicate significantly downregulated metabolites. **(F)** Distribution of up- and down-regulated differential metabolites across chemical classes.

Since the colon holds hundreds of metabolites involved in the orchestration of immune response and inflammation, then the widely-targeted metabolomics was performed to investigate the alteration of gut metabolites upon DCA treatment. Principal component analysis (PCA) and cluster analysis revealed significant sample clustering between the control and DCA groups (Figures 3A,B). Metabolites across 25 classes were identified, involving amino acids, nucleotides, organic acids, fatty acids, alcohol and amines, bile acids, CoEnzyme and vitamins, steroid and phenolic acid (Figure 3C). Heat map displayed a pattern of intra-group aggregation and inter-group segregation, indicating pronounced differences in the metabolite components between the two groups (Figure 3D). Fold change (FC) > 2 and *p* < 0.05 as the threshold, volcano plot based on metabolomics data showed that a total of 129 differentially expressed metabolites (DEMs) were identified in the comparison between CON and DCA groups, including 53 upregulated metabolites and 76 downregulated metabolites (Figure 3E). Majority of the altered metabolites belonged to organic acid and its derivatives, benzene and substituted derivatives, fatty acids, bile acids, amino acid and its metabolites by KEGG classification (Figure 3F). Further KEGG pathway enrichment analysis exhibited that retrograde endocannabinoid signaling, bile secretion, vitamin digestion and absorption, thermogenesis, fat digestion and absorption were the main significantly enriched KEGG pathways in response to DCA treatment (data not shown). These data suggested that excessive DCA reshaped the gut metabolome.

### 3.3 DCA reshapes gut microbiota-metabolic network

**Figure 4:**
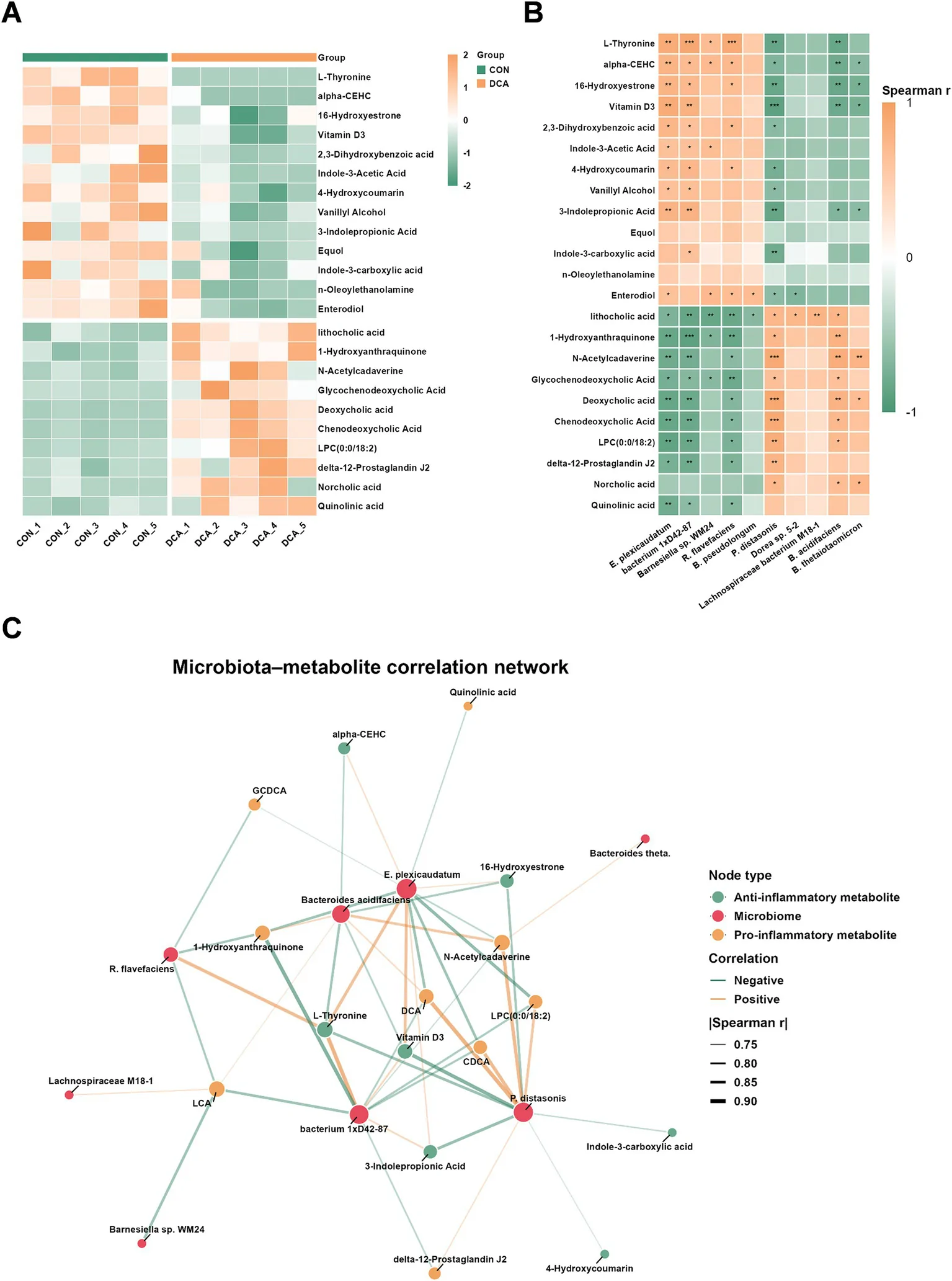
DCA reshapes gut microbiota-metabolic network. **(A)** Heatmap clustering of inflammation-associated differentially expressed metabolites between control and DCA groups. **(B)** Heatmap of Spearman’s correlation between the abundance of the 10 candidate differential microbial species and inflammation-associated differential metabolites in control and DCA groups. The statistical significance was shown inside the squares. **(C)** Co-occurrence network analysis of candidate microbial species and pro-inflammatory/anti-inflammatory metabolites. |Spearman *r*| ≥ 0.75 and *p* < 0.05. *p < 0.05; **p < 0.01; ***p < 0.001.

Based on previous published inflammation-related studies and the pro-inflammatory phenotypes of DCA administration, 23 DEMs were identified as potential candidates relevant to DCA-associated intestinal inflammation, including 13 anti-inflammatory related metabolites whose levels decreased upon DCA exposure, as well as 10 pro-inflammatory metabolites whose abundance increased following DCA intervention (Figure 4A). Further microbiota-metabolite correlation analysis revealed two opposing association modules between core microbiota and candidate differential metabolites. Microbial species significantly reduced in the DCA group, especially *E. plexicaudatum*, *bacterium 1xD42-87* and *R. flavefaciens*, were primarily positively correlated with anti-inflammatory related metabolites. Conversely, *P. distasonis* and *B. acidifaciens* which increased in the DCA group, were mainly positively correlated with pro-inflammatory related metabolites (Figure 4B). Microbiota-metabolite correlation network (|Spearman r| ≥ 0.75 and *p* < 0.05, Figure 4C) also supported that *E. plexicaudatum*, *B. acidifaciens*, *P. distasonis*, *bacterium 1xD42-87* and *R. flavefaciens* served as the major hub taxa, among which *B. acidifaciens* and *E. plexicaudatum* exhibited strong correlation with multiple inflammation-related metabolites. These data indicate that excessive DCA resulted in a significant reprogramming of the gut metabolome, which may be achieved through reshaping of the microbiota-associated metabolic network.

### 3.4 DCA administration is associated with increased intestinal inflammation

**Figure 5:**
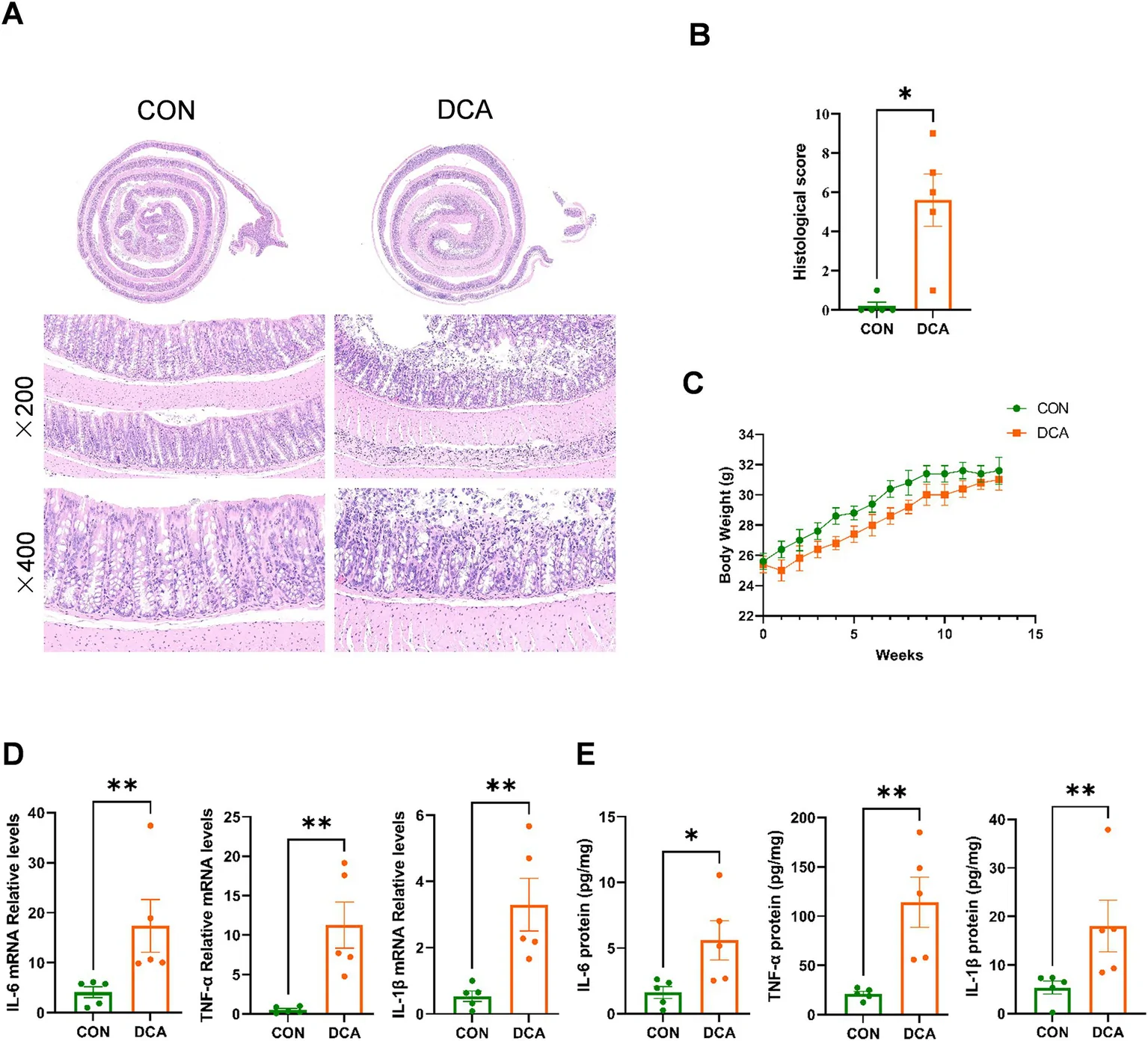
DCA administration is associated with increased intestinal inflammation. **(A–C)** Representative HE staining of colon tissue sections **(A)**, histological scores of colon tissue from CON and DCA-administered mice **(B)**, and **(C)** body weight. **(D)** Real-time PCR, and **(E)** ELISA analysis of IL-6, TNF-α, and IL-1β levels of colonic homogenates (*n* = 5 per group). *p < 0.05; **p < 0.01 compared to the control mice. Data are expressed as mean ± s.e.m.

HE staining of colonic tissue sections and pathological scoring exhibited that DCA administration was associated with obvious inflammatory cells infiltration and tissue damage in colon (Figures 5A,B). Body weight was monitored weekly and results showed that DCA exposure had no remarkable impact on the weight gain of mice in comparison with the control group (Figure 5C). However, significantly increased mRNA and protein expression of multiple pro-inflammatory cytokines, including IL-1β, TNF-*α* and IL-6, was observed in the colon tissue of DCA-treated mice compared with the counterparts in the control group (Figures 5D,E). These data indicate that excessive DCA indeed correlates with the occurrence of intestinal inflammation.

### 3.5 Integrated correlation analysis linking gut microbiota, critical metabolites, and inflammatory markers under DCA exposure

**Figure 6:**
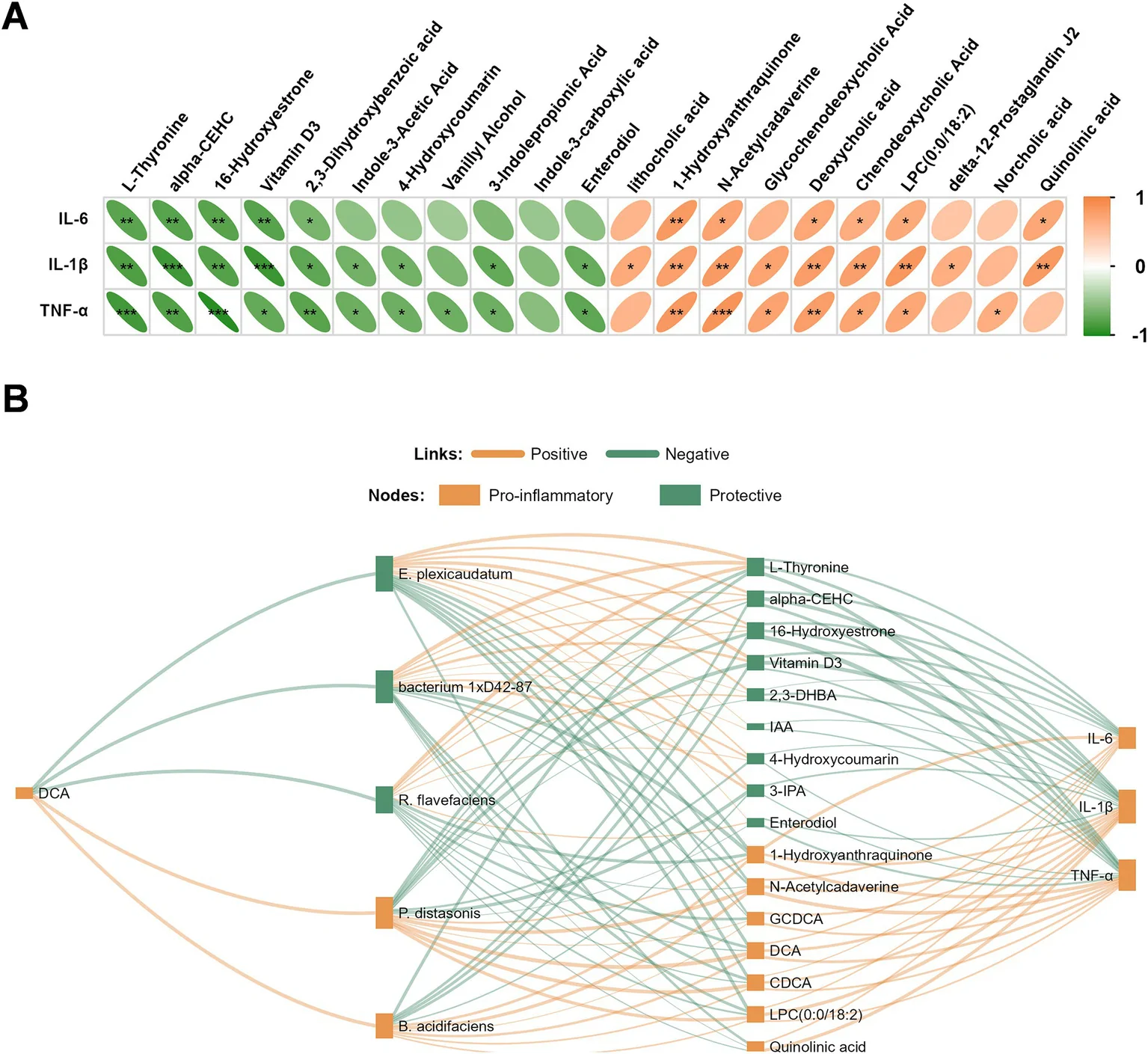
Integrated correlation analysis linking gut microbiota, critical metabolites, and inflammatory markers under DCA exposure. **(A)** Correlation analysis between differential metabolites and inflammatory cytokines. **(B)** Sankey plots showing the correlation among DCA, gut microbiota, fecal metabolites, and inflammatory cytokines. The thickness of the line is proportional to the correlation degree. |Spearman *r*| ≥ 0.30 and *p* < 0.05. *p < 0.05; **p < 0.01; ***p < 0.001.

To confirm the association of the core microbiota, candidate differential metabolites and the colonic inflammation upon DCA challenge, spearman correlation analysis was performed. Correlation analysis between candidate metabolites and colonic inflammatory cytokines in our DCA exposure model showed that metabolites reported to have anti-inflammatory activities, especially Vitamin D3, alpha-CEHC, L-Thyronine, 16-Hydroxyestrone and 2,3- Dihydroxybenzoic acid, were inversely correlated with IL-6, IL-1β and TNFα, while metabolites reported to possess pro-inflammatory properties, particularly 1-Hydroxyanthraquinone, N-Acetylcadaverine, DCA, CDCA were positively correlated with the inflammatory cytokines (Figure 6A). Subsequently, metabolites that exhibited significant correlations with at least two pro-inflammatory markers were selected for further Sankey diagram visualization. Sankey diagram integrated the associations among DCA, core microbiota, candidate metabolites and inflammatory markers (|Spearman r| ≥ 0.30 and *p* < 0.05, Figure 6B). DCA exposure upregulated the abundance of pro-inflammatory-associated microbiota (*B. acidifaciens* and *P. distasonis*), which were positively correlated with pro-inflammatory-related metabolites (bile acids, lipids and polyamine) and subsequently inflammatory cytokines. Meanwhile, DCA exposure downregulated the abundance of protective microbiota (*E. plexicaudatum*, *bacterium 1xD42-87* and *R. flavefaciens*) and consequently correlated with the reduction of anti-inflammatory-related metabolites (indoles, vitamin and hormone), which negatively associated with the levels of inflammatory markers. These results suggest that amplified pro-inflammatory microbial-metabolic pathway combined with the attenuated anti-inflammatory microbial-metabolic pathway may synergistically contribute to the DCA-associated intestinal inflammation. Therefore, identification of key metabolites may provide new targets for the management of HFD-associated colonic inflammation, which requires validation through further functional experiments.

## 4 Discussion

HFD-associated high level DCA in intestine is closely related to the colonic inflammation and increased risk for IBD. However, the factors participate in the DCA-associated gut inflammation still need to be clarified. Since gut microbiota and multiple metabolites are believed to play critical roles in intestinal homeostasis and inflammation, in this study, we compared the gut microbiota profile and fecal metabolites in control and DCA administrated mice. Our findings showed that excessive DCA reduced microbial diversity, altered gut microbiota composition, and disrupted fecal metabolome profile. Notably, a significant increase in the *P. distasonis* and *B. acidifaciens* species, accompanied with a pronounced decrease in the *E. plexicaudatum*, *bacterium 1xD42-87* and *R. flavefaciens* species were observed in response to DCA treatment, which correlated with the elevation of bile acid metabolites as well as the reduction of indoles, vitamin and hormone-like metabolites in the feces. Spearman correlation analysis emphasized the important effects of above mentioned microbiota and metabolites in the association between DCA and intestinal inflammation. Therefore, these data provide a potential mechanism that high level DCA is associated with intestinal inflammation through remodeling of gut microbiota-metabolite system.

In addition to promoting fat digestion and regulating metabolic signals, the association between DCA and inflammation has been gradually recognized. Previous studies mainly focused on the direct effects of excessive DCA on the mucosal immune system, recently, increasing evidence suggest that gut microbiota and metabolites may play crucial mediating roles in the initiation and development of intestinal inflammation (Wang J. et al., 2024; Yang et al., 2026; Oleszycka et al., 2023; Zhang et al., 2023). The interaction between bile acid and microbiota has been well accepted. High-fat diet increases the abundance of key intestinal bacteria involved in the synthesis of SBAs in the colon (such as Clostridium clusters XI and XIVa) (Yoshimoto et al., 2013), resulting in the significant elevation of SBA levels, particularly DCA. Thus, gut microbiota could modulate bile acids pool. Meanwhile, bile acids can also affect the growth and diversity of the gut microbiota, and the dysregulation of bile acid-gut microbiota system may lead to various diseases. For instance, taurocholic acid could stimulate the growth of *Bilophila wadsworthia*, which inhibits butyrate oxidation through generating hydrogen sulfide, thus interrupting energy balance in enterocytes and eventually leading to gut leakage and inflammation (Devkota et al., 2012). Since a significant increment of DCA has been observed under HFD conditions, here we explored the impact of high level DCA on the structure of intestinal flora. Abundance analysis together with LEfSe suggested that *Bacteroides* and *Desulfovibrio* represented potential biomarkers of DCA exposure. Correlation analysis found that *Bacteroides* abundance was positively associated with colonic IL-6 (*ρ* = 0.842, *p* = 0.0037, *q* = 0.0216), IL-1β (ρ = 0.806, *p* = 0.0072, *q* = 0.0216) and TNF-*α* (ρ = 0.758, *p* = 0.0149, *q* = 0.0299), while *Desulfovibrio* showed weaker positive associations with these three inflammatory markers (data not shown). However, more published functional evidence links *Desulfovibrio* to intestinal barrier dysfunction and inflammation. As sulfate-reducing bacteria, *Desulfovibrio* has been reported to increase tight junction permeability through a Snail-dependent pathway (Singh et al., 2022), and the hydrogen sulfide they generate can compromise mucus integrity and epithelial metabolism (Ijssennagger et al., 2016). Administration of *Desulfovibrio vulgaris* causes gut inflammation and aggravates DSS-induced colitis in mice (Huang et al., 2024). Furthermore, previous research suggests a plausible ecological connection between these two genera. *Bacteroides* species encode extensive sulfatase repertoires that liberate sulfate from host mucin and glycosaminoglycans (Ulmer et al., 2014; Luis et al., 2021). Co-culture study has shown that *B. thetaiotaomicron* enhances hydrogen sulfide production by a sulfidogenic partner organism (Davies et al., 2024). This offers a possible explanation for the co-enrichment of these two genera. Additionally, it is known that DCA mainly inhibited Gram-positive bacteria by disrupting bacterial membrane integrity, consistently, we found that DCA administration significantly reduced the abundance of *E. plexicaudatum*, *bacterium 1xD42-87* and *R. flavefaciens*, all of which belong to the phylum *Firmicutes* (Gram-positive). *E. plexicaudatum* and *R. flavefaciens* are previously considered beneficial in part due to their association with enhanced short chain fatty acid (SCFA) production (Breyner et al., 2019; Sánchez-Tapia et al., 2020). Importantly, our findings disclosed that down-regulation of *E. plexicaudatum*, *bacterium 1xD42-87* and *R. flavefaciens* induced by DCA were positively correlated with the reduction in known anti-inflammatory indoles, vitamin and hormone metabolites, which provide possible explanations for the anti-inflammatory mechanisms of aforementioned bacteria (Li M. et al., 2024; Zhou et al., 2024; Himmelfarb et al., 2003). Of Note, *bacterium 1xD42-87* currently still belongs to a bacterial species not fully classified, although it exhibited protective tendency against gut inflammation based on our data. Furthermore, we observed that excessive DCA simultaneously increased the proportion of *P. distasonis* and *B. acidifaciens*, which mainly correlated with the remodeling of bile acid metabolism, as evidenced by the general elevation of CDCA, GCDCA and LCA in feces that largely exhibit pro-inflammatory properties at high levels (Liu et al., 2022; Zeng et al., 2019; Oleszycka et al., 2023). Thus we proposed a gut microbiota-metabolism imbalance pattern associated with DCA-related inflammation. Specifically, DCA treatment enhances certain pro-inflammatory microbiota-metabolic axes, and concomitantly it impairs several anti-inflammatory microbiota-metabolic barriers, which may collectively contribute to the development of intestinal inflammation.

Notably, DCA has bidirectional biological effects, and it exhibits pro-inflammatory or anti-inflammatory activities depending on experimental settings. Dose is a critical determinant that high dose of DCA commonly correlates with the pro-inflammatory effect. Increasing DCA exposure perturbs intestinal epithelial cell membranes, induces reactive oxygen species (ROS) production, and disrupts tight junction integrity, thereby increasing intestinal permeability (Stenman et al., 2013a; Zeng et al., 2022). In addition, DCA dose-dependently activates NLRP3 inflammasome in macrophages and promotes Th17 differentiation, thus driving the production of diverse pro-inflammatory cytokines (Zhao et al., 2016; Li et al., 2023). Conversely, at physiologically relevant lower concentrations, DCA may elicit anti-inflammatory or homeostatic responses. For instance, restoration of DCA deficiency has been shown to ameliorate DSS-induced colitis partly through TGR5-dependent signaling (Sinha et al., 2020). In addition to dosage, exposure duration shapes phenotypic outcomes. It has been reported that inflammation and barrier-related transcriptional changes were prominent at 1 month after sustained DCA treatment, which may reflect an early acute response, and persisted thereafter that may lead to chronic inflammatory activation and mucosal damage (Bernstein et al., 2006). The route of DCA administration strongly influences local bioavailability which also accounts for the different biological effects. Dietary admixture, continuous drinking-water administration and colorectal instillation differ in the duration, thus yielding different temporal patterns of bile acid exposure. Enema delivery is typically used in the mouse model of experimental colitis induced by DSS or TNBS to evaluate the protective or exacerbating effects of DCA on colitis (Sinha et al., 2020; Zhao et al., 2016). While Dietary admixture or continuous drinking-water administration is commonly used to simulate the effect of prolonged HFD exposure (Bernstein et al., 2006; Pan et al., 2024). In addition, the accurate DCA concentration reached in the tissue is ill-defined. The administered DCA dose does not directly represent the actual concentration in intestinal lumen or mucosal tissues, which is due to *in vivo* metabolism, bacterial transformation and bile acid reabsorption (Stenman et al., 2013a). Furthermore, the baseline disease context of experimental animals, including pre-existing gut injury and genetic susceptibility, can further modify DCA-mediated phenotypes. In chemically induced colitis models, DCA administration significantly exacerbates DSS- and TNBS-induced intestinal inflammation (Zhao et al., 2016; Wang C. et al., 2024). Similarly, in genetically predisposed Apc^min/+^ mice, chronic DCA exposure induces low-grade gut inflammation and promotes intestinal tumorigenesis (Liu et al., 2018). Collectively, multiple variables may account for the divergent outcomes of DCA exposure. A systematic dose–response and time-course study with quantification of luminal and mucosal DCA will be implemented in our follow-up research.

Amino acid metabolism plays an important part in gut homeostasis, and multiple specific derivatives of amino acid transformed by microbiota can serve as signaling molecules with protective or detrimental effects. Here we found that idoles, as the major products of microbial tryptophan metabolism, including idole-3-acetic acid, indole-3-propionic acid and indole-3-carboxylic acid, were significantly down-regulated upon DCA exposure. The anti-inflammatory role of indoles has already been extensively investigated and a significant reduction of fecal idoles has been observed in patients with IBD (Schroeder and Bäckhed, 2016; Lee et al., 2015). Mechanistically, indoles could inhibit inflammation through inducing the expression of anti-inflammatory cytokine IL-10, while decreasing TNF-*α*-mediated NF-κB activation (Gao et al., 2018). Moreover, indoles promote the expression of tight junction proteins of intestinal epithelium, and thereby protecting the intestinal mucosal barrier (Alexeev et al., 2018). Our findings suggest that DCA exposure-associated down-regulation of idoles in feces may participate in the development of gut inflammation, while supplementation with the tryptophan idoles metabolites could be a potential management for HFD-associated inflammation.

An intriguing finding of our study is that vitamin D3 was dramatically down-regulated upon DCA treatment. Vitamin D is the general name for vitamin D3 and vitamin D2, among which vitamin D3 is the main component with the highest bioavailability. Previous epidemiological studies have revealed that levels of vitamin D were inversely related to IBD, including Crohn’s disease (CD) and ulcerative colitis (UC), and more than half of the patients were deficient in vitamin D (Li et al., 2019; Basson, 2014; Wang H. et al., 2022). Moreover, subnormal levels of vitamin D positively correlates with disease severity of CD and UC (Ko et al., 2019; Blanck and Aberra, 2013). Mechanistic research has shown that vitamin D3 could inhibit inflammation partially through regulating NLRP6 and NLRP3 inflammasome activation, in addition, ameliorate intestinal tissue injury via promoting colonic epithelial proliferation and maintaining mucosal barrier integrity (Gao et al., 2023; Cao et al., 2020; Kellermann et al., 2024). So far as HFD is concerned, it commonly leads to the decrease of vitamin D concentrations, and dietary-associated vitamin D3 insufficiency consequently exacerbates intestinal inflammatory responses to infection (Ryz et al., 2015). All these studies indicate that the decrease of vitamin D makes great contribution to the HFD-induced gut inflammation. However, HFD-related drivers and potential mechanisms underlying vitamin D deficiency remain unclear. Our findings in this study showing that excessive DCA closely correlated with the reduced intestinal vitamin D3 levels, together with the fact that vitamin D3 levels exhibited strong negative correlation with the inflammatory cytokines, these data highlighted the importance of DCA-vitamin D3 axes in the association with HFD-induced gut inflammation. Another vitamin-related metabolite, namely *α*-CEHC, was also significantly reduced in DCA-treated group. α-CEHC is identified as the terminal water-soluble degradation product of α-tocopherol, representing the responsive biomarker of vitamin E metabolism (Leonard et al., 2005). Compared to the weak anti-inflammatory effect of α-tocopherol, α-CEHC has been proved to possess strong anti-inflammatory ability (Grammas et al., 2004). Importantly, vitamin E is reduced in patients suffering from Crohn’s disease, meanwhile, decreased concentrations of α-CEHC glucuronide have been observed in the urine of IL10^−/−^ mice, which was regarded as a promising marker of intestinal inflammation (Kuroki et al., 1994; Otter et al., 2011). Therefore, the down-regulation of α-CEHC under DCA exposure may also be an possible mechanism involved in HFD-associated inflammation. Additionally, gut microbiota such as *E. plexicaudatum*, *bacterium 1xD42-87* and *R. flavefaciens* may play certain role in mediating DCA-associated vitamin D3 and α-CEHC reduction, which need exploration in the further studies. It is noted that the alteration of vitamin D3, indoles, and α-CEHC are not exclusively governed by gut microbiota. Vitamin D homeostasis is also controlled by dietary intake, intestinal absorption, and hepatic and renal metabolism. Intestinal indole levels depend on dietary tryptophan and intestinal epithelial transport in addition to microbial metabolism. Similarly, α-CEHC abundance is affected by dietary vitamin E supply and hepatic metabolism together with gut microbial transformation. Therefore, the reduction of these metabolites in the current model cannot be solely attributed to DCA-mediated gut microbiota alteration. Direct causal relationships would require further interventional experiments such as microbiota depletion.

Although our study integrates metagenomic sequencing and widely-targeted metabolomics to perform extensive analysis, some limitations still need to be considered. Our study supports the existence of a significant correlation network among DCA, gut microbiota, metabolites and inflammatory markers, however, definitive causal relationships between individual microbial taxa or specific metabolites and the observed intestinal inflammatory phenotypes cannot be strictly established. Meanwhile, the microbiota-metabolite axis is proposed in the present study as a potential indirect regulatory pathway of DCA-associated gut inflammation. Actually, DCA may exert pro-inflammatory effects through two potential pathways: (i) direct effects on immune cells and intestinal epithelial cells; (ii) indirect pathway via reshaping gut microbiota and downstream metabolites. Numerous studies have focused on the direct effects of excessive DCA on mucosal immune system. Our observations here provide preliminary correlative evidence suggesting the hypothetical microbiota-metabolite-mediated indirect pathway. Future functional intervention studies, such as antibiotic-mediated microbiota depletion, fecal microbiota transplantation, DCA inhibition, or exogenous metabolite supplementation, are required for separating DCA direct effects from microbiota-mediated indirect effects, validating biological roles of candidate metabolites in the DCA-associated colonic inflammation, and verifying our mechanistic hypotheses. Moreover, sex-associated differences in hormone and lipid metabolism should not be neglected. The present study only included male mice, which also restricts the comprehensiveness of the research. Future analysis with both genders would be better to elucidate critical gut microbiota and metabolites mediating the effects of DCA on gut inflammation.

In conclusion, our study found that DCA exposure resulted in concurrent alterations of gut microbiota and metabolites, certain of which showed significant correlations with intestinal inflammation. Considering the importance of dietary factors on the inflammatory diseases, our findings suggest that targeting DCA related gut microbiota or metabolites may be potential approaches for HFD-induced colitis.

## Data Availability

The raw metagenomic sequencing data generated in this study are publicly available in the NCBI Sequence Read Archive (SRA) under BioProject accession PRJNA1478809.
